# Impact of a recognition package as an incentive to strengthen the motivation, performance, and retention of village health teams in Uganda: a study protocol for a cluster randomized controlled trial

**DOI:** 10.1186/s13063-023-07426-6

**Published:** 2023-06-23

**Authors:** Smisha Agarwal, Raymond Tweheyo, Shivani Pandya, Emmanuel Obuya, Arisa Kiyomoto, Paloma Mitra, Meike Schleiff, Tanvi Nagpal, Mario Macis, Elizeus Rutebemberwa

**Affiliations:** 1 The Johns Hopkins Bloomberg School of Public Health, MD Baltimore, USA; 2 Makerere University School of Public Health, Kampala, Uganda; 3 The Johns Hopkins University Krieger School of Arts & Sciences, Baltimore, MD USA; 4 The Johns Hopkins University School of Advanced International Studies, Foreign Policy Institute, Washington, DC USA; 5 The Johns Hopkins University Carey School of Business, Baltimore, MD USA

**Keywords:** Community health workers, Community health systems, Incentives, Motivation, Performance

## Abstract

**Introduction:**

Uganda’s community health worker (CHW), or village health team (VHT), program faces significant challenges with poor retention and insufficient financial and program investment. Adequate compensation comprising financial and non-financial components is critical to retaining any workforce, including CHWs. This study evaluates the impact of a recognition-based non-financial incentives package on the motivation, performance, and retention of VHTs, as well as on the utilization of health services by the community. The incentive package and intervention were developed in collaboration with the district-level leadership and award VHTs who have met predetermined performance thresholds with a certificate and a government-branded jacket in a public ceremony.

**Methods:**

A two-armed cluster randomized controlled trial (RCT), conducted at the parish level in Uganda’s Masindi District, will evaluate the effects of the 12-month intervention. The cluster-RCT will use a mixed-methods approach, which includes a baseline/endline VHT survey to assess the impact of the intervention on key outcomes, with an expected sample of 240 VHTs per study arm; our primary outcome is the total number of household visits per VHT and our multiple secondary outcomes include other performance indicators, motivation, and retention; VHT performance and retention data will be validated using monthly phone surveys tracking key performance indicators and through abstraction of VHT-submitted health facility reports; and focus group discussions will be conducted with VHTs and community members to understand how the intervention was received. Data collection activities will be administered in local languages. To assess the impact of the intervention, the study will conduct a regression analysis using Generalized Estimating Equations adjusting for cluster effect. Further, a difference-in-differences analysis will be conducted.

**Discussion:**

This study utilized a cluster-RCT design to assess the impact of a recognition-based incentives intervention on the motivation, performance, and retention of VHTs in Uganda’s Masindi District. Utilizing a mixed-methods approach, the study will provide insights on the effectiveness and limitations of the intervention, VHT perspectives on perceived value, and critical insights on how non-financial incentives might support the strengthening of the community health workforce.

**Trial registration:**

ClinicalTrials.gov NCT05176106. Retrospectively registered on 4 January 2022.

**Supplementary Information:**

The online version contains supplementary material available at 10.1186/s13063-023-07426-6.

## Background

Community health workers (CHWs) play a critical role in strengthening primary healthcare (PHC) globally—especially in rural areas, where there is often a shortage of skilled healthcare workers (i.e., physicians, nurses, and midwives, etc.). In Uganda, approximately 75% of the population is rural, while the majority of healthcare workers are concentrated in urban areas [1]. Uganda’s CHW program was launched in 2001 and has been scaled nationally—operating in all of Uganda’s 135 districts [2, 3]. Their CHW program comprises village health workers (VHWs) who work in teams of 5–6 people, which are known as “village health teams” (VHT) [2]. VHTs are a voluntary cadre, elected by the community members of their resident communities which they serve [2]. VHTs provide maternal, newborn, child health, and sanitation-related services. Since 2001, the VHT program has demonstrated positive impacts on health outcomes, particularly for communities that would otherwise lack access to health services [4–7]. Between 2001 and 2015, over 179,000 individual VHTs have been trained [3]. Like several CHW programs globally, the VHT program has faced many challenges due to high levels of attrition [8]. Lack of adequate compensation for their work and ongoing training, supervision, logistical, and financial support, as well as threats to personal health (e.g., COVID-19 pandemic, Ebola), are some of the core reasons why this cadre of health workers, which is so critical to the health of rural populations, experiences high rates of attrition [3].

The lack of remuneration for CHWs has been a longstanding debate within the field, with a growing push to compensate them fairly [9]. The 2018 *WHO Guideline on health policy and system support to optimize community health workers program* recommended remunerating CHWs with a “financial package commensurate with the job demands, complexity, number of hours, training, and roles that they undertake” [10]. In addition to financial remuneration, the WHO guideline recommends the provision of non-monetary incentives to further support and motivate CHWs [10]. In Uganda, the 2014 National VHT Assessment, which assessed the status and functionality of the VHT program, reported on the importance of providing VHTs with a “standardized and harmonized regular and equitable financial form of motivation” as well as the “equitable distribution of non-monetary incentives” [11]. Despite these recommendations, much progress is still to be made regarding the appropriate and equitable compensation (financial and non-financial) for VHTs.

A number of studies have assessed the effectiveness of a variety of non-financial and financial incentives on the performance and impact of CHW programs across settings with mixed results [10]. A cluster randomized controlled trial (RCT) which evaluated a non-monetary incentives intervention in India found that the provision of public recognition (through a ceremony and certificate) resulted in greater CHW motivation than the other non-monetary incentives provided [12]. In Uganda, one study found that the provision of non-financial incentives to CHWs—t-shirts, umbrellas, gumboots, and certificates, which were provided to all CHWs—resulted in improved CHW performance and motivation [13]. A 2021 systematic review on performance-based incentives reported that the provision of public recognition for CHW efforts resulted in increased and improved service delivery [14]. In Guinea-Bissau, CHWs received a certificate for good performance at a public ceremony; results from this study found increased satisfaction with CHWs by the community as well as increased community knowledge around health practices [15]. In contrast to these results, a 2021 study in Uganda that evaluated the effects of a competitive social reward (referring to a type of reward that provides public recognition) provided to the best-performing CHWs found a negative association between CHW performance and the provision of the social reward. The study provided a reward to 3% of the 4050 CHWs over the 3-year period [16]. The authors hypothesized that the negative results might be a consequence of the design of the award mechanism which has subjective eligibility criteria leading to perceptions of favoritism, infrequent award provision, and inadequate transparency regarding the selection process [16]. Despite this, the authors affirm that the provision of a recognition-based performance award can be effective towards improving CHW performance, with an emphasis placed on a transparent and fair award provision process [16]. None of these studies evaluated the effects of their intervention on CHW retention and attrition. Our current study builds on this evidence to assess the effectiveness of a package of non-financial recognition-based incentives on the performance, motivation, and retention of VHTs in Uganda. This study also builds on prior studies conducted in collaboration with the Johns Hopkins Bloomberg School of Public Health, Population Council and Pathfinder International, Uganda using a discrete choice experiment (DCE) study to understand incentive preferences of VHTs in Uganda [17]. The DCE study identified a variety of incentives desired by VHTs. In this study, in collaboration with district-level partners, we further define these incentives and prioritize those that are not only valued by VHTs but are also pragmatic and sustainable for government uptake. This protocol was developed in adherence to the SPIRIT checklist for reporting on protocols in clinical trials (see Additional file 1) [18].

The objectives of the present study are to better understand (1) the effectiveness of recognition-based non-financial incentives, to support VHT motivation, performance, and retention; (2) the behavioral mechanism through which new incentives may succeed or fail; and (3) the impact of improved VHT performance on the utilization of maternal and child health services, hygiene practices, and perceptions regarding the quality of health services at the community level.

## Methods/design

We will conduct a two-armed cluster randomized, controlled superiority trial to evaluate the effects of the intervention on the motivation, performance, and retention of VHTs in Uganda’s Masindi District. The intervention comprises a recognition-based non-financial incentives package, designed in August 2021 in collaboration with local leadership from Masindi District; this process is described in further detail below.

### Study setting and participants

**Fig. 1:**
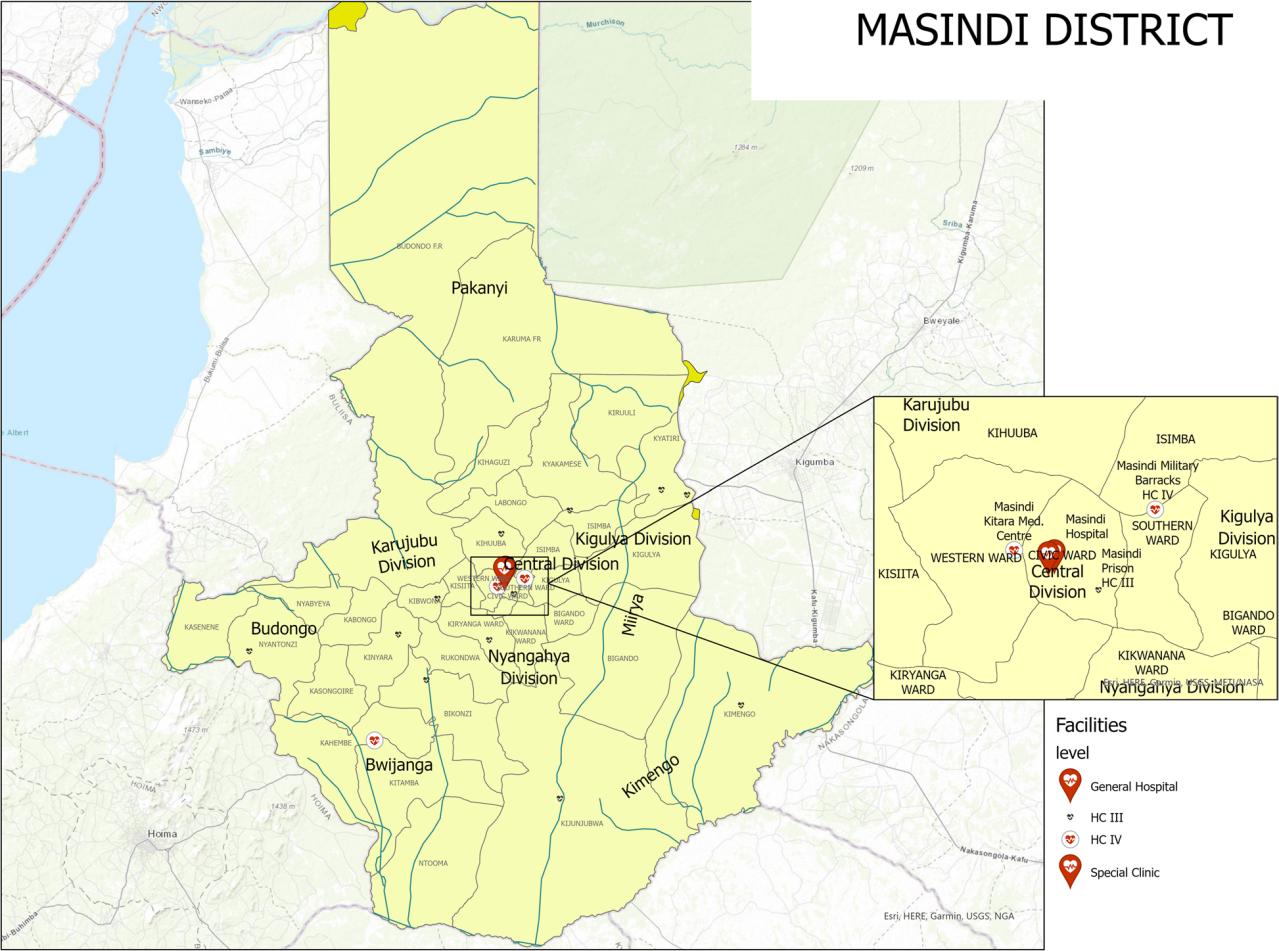
Masindi District, Uganda (July 2021)

The study will be implemented in Uganda’s Masindi District (see Fig. 1). As of July 2021, Masindi District has nine sub-counties, 32 parishes, and 312 villages. Masindi is a predominantly rural district, with most of the population living far from the main roads. Around 30% of the population lives on less than $1 per day, per a 2012 estimate [19]. Compared to national averages, Masindi District has a higher maternal mortality rate (499 per 100,000 live births, as compared to 435 per 100,000) and a higher proportion of adolescent deliveries (24.8% compared to 20%) [20] compared to the national rates. Vaccination coverage is lower than the national average, with around 71.8% of children receiving the DPT3 vaccine on schedule, compared to 84% nationally [20]. The District also experiences a high prevalence of malnutrition and infectious and communicable diseases [20]. In 2020–2021, Masindi District was ranked 107th out of 136 districts per a composite index calculated by Uganda’s MoH which ranks districts from best to worst-performing, signifying poor health sector performance [21]. This index considers staffing levels; coverage (e.g., tuberculosis case notification rate, antenatal care 4th visit coverage, the proportion of deliveries in health facilities, etc.); quality of care (e.g., the proportion of maternal deaths that are reviewed, the proportion of perinatal deaths that are reviewed, etc.); community health services (e.g., community VHT quarterly reporting rate, the proportion of children under-five dewormed in last six months); and management (e.g., local government performance assessment score, supervision performance assessment and recognition strategy score) [21]. Per this index, the national average is 64.4%; Masindi District is at 57.3% [21], which indicates that Masindi District is in the lower 50th percentile of districts for critical health and sanitation parameters. Masindi District’s selection as the study site was guided by the poor coverage of maternal and child health outcomes, and the topographical diversity within the three distinct ecological zones, which allows for the assessment of the VHT program under a range of geographical conditions.

### Inclusion criteria

VHTs who are identified as active by the parish supervisors are eligible for enrollment in the study, following consent. As of 2020, there was an estimated 517 VHTs across 32 parishes in the district, with an average of 16.1 VHTs per parish. The study plans on recruiting all active VHTs.

### Randomization and blinding

The intervention will be randomized at the parish level. Parishes will be randomly assigned with equal probability to either the intervention or comparison group within each sub-county. All 32 parishes will be randomized. Randomization will be conducted by a biostatistician independent of the study team through random number generation using STATA [22]. The study team will be blinded from the process of allocation assignment. The random numbers will be generated uniformly, then ordered ranks will be generated separately with a rank order of 0 or 1. Rank 0 will be labeled the comparison parish and rank 1 will be labeled the intervention parish. Rank orders will then be randomly assigned to parishes. There will be 16 parishes/arm and approximately 259 VHTs in each arm.

The intervention will not be blinded to the study team as the study team is engaged in supporting the delivery of the intervention to support the Masindi district. The intervention will not be blinded to the participants as the intervention involves public recognition of high-performing VHTs.

### The intervention

The development of the intervention incentive package is guided by the insights gained through a prior study that used a discrete choice experiment to understand the incentive preferences of the CHWs in Uganda [17]. The study indicated that reliable transportation (such as bicycles) and recognition (through the use of branded uniforms and/or identification cards) were highly valued by the CHWs. CHWs were willing to accept a decrease in salary of Ush 31,240 (US $8.5) for identity badges and of Ush 85,300 (US $23) for branded uniforms to no form of identification [17].

**Fig. 2:**
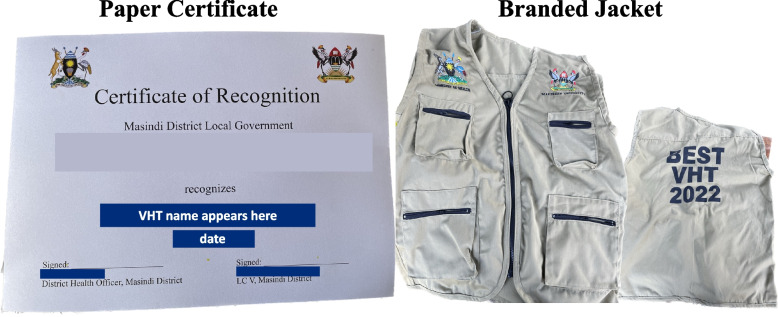
The recognition-based incentives provided during the public ceremony to VHTs

To determine the intervention to be tested and the criteria for assessing performance, the study team met with 16 stakeholders in Masindi District in August 2021. The stakeholders included VHT/Parish Coordinators; Health Assistants, who supervise VHTs; Health Inspectors; District Health Educators; District Health Officer; Biostatistician; and representatives/focal persons for district quality improvement, district surveillance, HIV, and malaria programs. The results of the prior study were presented to the stakeholders, and based on considerations of the sustainability of the intervention and the ability of the local district to financially sustain the intervention, the proposed intervention was adapted for the context. These discussions resulted in the following recognition-based incentives package: (1) a certificate which includes the name of the VHT and language describing the VHTs’ exemplary performance; (2) a branded jacket that would identify the VHT as a high performer (see Fig. 2); and a (3) a public recognition of VHTs who have been actively serving their communities.

All VHTs who have submitted their quarterly reporting forms (see Additional file 2) to the health facility will be considered for recognition. VHTs will be provided with regular reminders to submit quarterly reports to health facilities during VHT engagement activities (e.g., monthly VHT phone survey, health facility data abstraction). VHTs will be assigned to different strata based on the size of the rural/urban parishes (i.e., urban small, rural small, rural large) given the differences in the number of households and proximity of households. Finally, the top 10% of performers within each stratum will be publicly recognized. Performance of the VHTs will be assessed based on the following three indicators: the proportion of children under 5 years of age with up-to-date immunization, the proportion of sick children under 5 years of age the VHT has attended to, and the proportion of women who attended at least four antenatal care visits. These indicators were chosen as they are what VHTs record in and report from their registers and are likely to be achieved by VHTs. The denominators for each variable will be abstracted at the village level from the official population projection statistics provided by the Masindi District government, District Planner’s Office.

The public recognition ceremony will be convened on a quarterly basis, as the VHTs provide their activity reports on a quarterly basis. Representatives from the district will convene and lead the ceremony and all VHTs from the intervention parishes will be invited. For the comparison parishes, it will be standard practice, where VHTs continue to provide services as usual; there will be no public recognition ceremony for VHTs who are from the comparison parishes. Any changes to the intervention, if needed, will be made based on the input from the District; changes will be made at the intervention (status of randomization) level, and not at the VHT level, and will be documented for better contextual understanding. Intervention adherence will be facilitated by regular reminders to VHTs to submit quarterly reports to health facilities during VHT engagement activities (e.g., monthly VHT phone survey, health facility data abstraction).

### Primary and secondary study outcomes

This study utilizes a mixed-methods approach to understand the impact of the intervention on the primary and secondary outcomes. The primary outcome of this study is changes in VHT performance (defined as the total number of home visits in the past three months). VHT performance is challenging to assess accurately as often VHTs may not have the tools to record their visits (e.g., registers), may not have received training to correctly record the information, and may not maintain records to accurately recall their activities. Therefore, VHT performance data will be collected in three specific ways: (1) through VHT surveys conducted at baseline and endline; (2) through the abstraction of quarterly reported data submitted to health facilities as VHTs are expected to report quarterly; and (3) through monthly phone surveys. Secondary outcomes for this study include the following: (i) VHT motivation (assessed using the validated Close-to-Close (CTC) Provider Motivational Indicator Scale [23]); (ii) VHT retention (measured through monthly self-reports); (iii) the other VHT performance measured by trends in service delivery; and (iv) trends in adoption of sanitary practices in each village. See Table 1 for the list of primary and secondary outcomes and the related data collection activities to measure them.

**Table 1:** Primary and secondary outcomes and related data collection activities

	**Data collection activity**
**Primary outcome**	
** 1. VHT performance** *No. of total home visits in the last 3 months*	VHT survey VHT phone survey Abstraction from health facility report
**Secondary outcomes**	
** 2. VHT motivation** *Measured through the CTC Provider Motivational Scale. This scale has four sub-modules on work satisfaction, organizational commitment, community commitment, and work conscientiousness*	VHT survey
** 3. VHT retention** *Assessed through the proportion of leaving VHTs*	VHT phone survey
** 4. Other VHT performance (trends in service delivery)** *No. of visits for antenatal care* *No. of visits for postnatal care* *No. of visits to support immunization of children* *No. of referrals made to health facility* *No. of sick under-5 children attended by a VHT* *No. of people provided with HIV counseling* *No. of people provided with TB counseling* *No. of people provided with general counseling*	VHT survey VHT phone survey Abstraction from health facility report
** 5. Trends in adoption of sanitary practices per village** *No. of households with latrines* *No. of households with improved latrines* *No. of households with handwashing facilities* *No. of households with safe drinking water* *No. of households that are open defecation free*	VHT survey Abstraction from health facility report

### VHT recruitment and mobilization

The study team is working with the District to implement this study. Initial meetings were held to orient the District leadership to the study concept and study team. District leadership comprised the District Health Officers (DHO), District Focal Person for Community Health Activities, and the Biostatistician. The District Focal Person provided the study team with an orientation on VHT activities and responsibilities, as well as a list of currently active VHTs and Parish Coordinators. The study team will utilize this list to reach out to VHTs for recruitment and consent.

### Training data collectors

A 3-day training will be held to orient data collectors to the study and provide them with training on the study tools, human subjects research and ethics, and the consenting process. The third day of training will focus on using Open Data Kit (ODK) [24], a tablet-based data collection platform, and on pretesting the survey tool. All data collectors will have received a Bachelor’s degree and have fluency in Runyoro, Lugbara, and/or Kiswahili—the prevalent local languages in the study district.

### Data collection and timeline

**Fig. 3:**
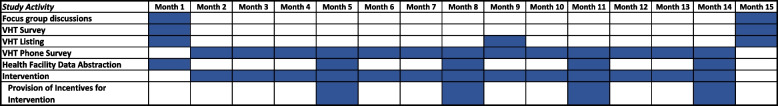
Timeline of study activities

Trained data collectors will implement the data collection activities. See Fig. 3 for the timeline of the intervention and data collection activities. To ensure participant retention for these data collection activities, the study team will provide regular reminders, which will occur via direct phone calls tied to upcoming data collection activity and at the quarterly recognition ceremonies.

#### VHT survey

A structured questionnaire will be administered to all VHTs at baseline and endline. The survey comprises the following modules: demographics, VHT experience (i.e., current engagement as a VHT, compensation, assets and other revenues, trainings, responsibilities), VHT performance (i.e., their activities and health services provided to the community over the last three months), VHT motivation, and COVID-19 experiences as a VHT. The VHT motivation module was adapted from the 12-item Close-to-Community Health Workers (CTC) Provider Motivational Indicator Scale and comprises four factors: work satisfaction, organizational commitment, community commitment, and work conscientiousness [23]. The COVID-19 module was developed by the study team and only included in the baseline. The survey will be implemented in three languages: Runyoro, Lugbara, and Kiswahili. Survey data will be digitally collected on tablets using ODK [24].

#### Health facility report abstraction

The study team will abstract service delivery records from the paper reports that VHTs submitted to health facilities to triangulate VHT performance data and capture trends in the delivery of health services. The reporting format is the HMIS VHT 097b: VHT/ICCM Quarterly Village Report (see Additional file 2). This will occur on a quarterly basis—at four points in time for the prior 3 months.

#### VHT listing

The study team will work with the VHT coordinator and health facility in charge to list out all active VHTs. This activity will also be used to gather and update contact details and phone numbers for the VHTs. This will occur at three timepoints: baseline, midpoint (6 months), and endline, and will serve to capture all active VHTs in the District.

#### VHT phone survey

All VHTs who have a phone will be administered a brief monthly phone survey to report on key performance indicators over the last month. The VHTs will be called by trained research assistants who are stationed in a call center in Kampala, Uganda. Calls will be made in local languages over the course of 4 days. The research assistant will make at least three attempts each month to reach the VHT. If the phone number is no longer active or connected to the VHT, the study team will reach out to the Parish Coordinator to connect with those VHTs. The questions are adapted from questions included in the VHT performance module in the VHT Survey. This data will be compared against the endline survey and quarterly-administered health facility reports to triangulate the findings. The survey will be administered using ODK [24].

#### Focus group discussions (FGDs)

FGDs will be conducted with VHTs and community leaders at baseline and endline. The goal of the FGDs with VHTs is to understand the type and structure of incentives that they prioritize. Community leaders will be engaged with the FGDs to provide an understanding of their relationship with VHTs as well as their perceptions of VHT activities in advancing health services. Between 9 and 10 FGDs will be held with VHTs and 9 and 10 with community leaders; there will be between 6 and 8 participants in each FGD. FGDs will continue until saturation is reached. Each FGD will have two trained facilitators: one will moderate the discussion and the other will take notes.

### Power estimation and sample size

The study, based on the recommendations of the District Health Office, will recruit all active VHTs in 32 parishes (i.e., 16 clusters per treatment arm), with an expected total of 259 VHTs in each arm and a mean cluster size of 16 VHTs per cluster. We assumed a 90% response rate, where we anticipate a total of 15 responding VHTs per parish. This results in 240 VHTs in each arm in our sample. We conducted a power analysis using the two-sample means test for cluster randomization with total household visits per VHT in the last 3 months as our primary outcome, using STATA [22, 25]. Based on numbers reported in past literature (Tweheyo R, Rutebemberwa R: Capacity and readiness of the community health workforce for providing voluntary family planning services in central and Western Uganda, forthcoming), we assumed a mean of 55 household visits per VHT with a standard deviation of 25 visits and used a conservative intra-class correlation of 0.2 for lay workers [26, 27]. The planned two-sided test aims significance level of 0.05 with a power of 0.8. Given these assumptions, our sample size is powered to detect an effect size of 12.5 household visits difference between the two arms.

### Patient and public involvement

FGDs held with VHTs and community leaders as well as collaboration with district-level leadership informed the incentives package and intervention design. Study results will be disseminated in collaboration with district-level leadership and policy-level stakeholders to further inform VHT policy in Uganda.

### Analysis plan

To assess the impact on the primary outcome (i.e., total household visits made per VHT), we will use Generalized Estimating Equations (GEE) to perform a linear regression analysis, adjusting for cluster effects at the parish level. A linear regression model assumes a normal distribution of the outcome: the number of household visits per VHT. If the data suggests that the outcome is not normally distributed, we will consider other model specifications such as the ordinal logit regression. The same approach will be taken for secondary outcomes to assess the estimated effects of the intervention. Intention-to-treat (ITT) with sensitivity analysis will be used to address protocol non-adherence. To clarify the ITT analysis approach, we plan to include all VHTs based on their allocation assignment at baseline data collection; however, if there are changes among VHTs in regard to their parish (i.e., if a VHT was initially in a comparison parish and has moved to an intervention parish during the duration of the study), we will monitor the percentage of VHTs that have crossed over (comparison to intervention parish and vice versa) and conduct sensitivity analyses to determine how to best proceed in regard to their inclusion in the analysis. We will document missingness patterns by intervention status to assess for random missingness. Depending on the type of missingness, we will consider strategies such as imputing or discarding the data. However, we do not expect the pattern to vary across the intervention and comparison arms.

Further, we will use a difference-in-differences (DiD) approach to control for baseline differences between the intervention and comparison groups as well as temporal differences that may have resulted from underlying changes over time. Finally, a sub-group analysis by VHT demographic characteristics and baseline performance scores will also be completed to examine the effect modification on the outcome. For the qualitative data collection (i.e., FGDs), transcripts will be reviewed by the study team. The study team will develop a codebook using a combination of deductive and inductive approaches to reflect the study’s objectives and address any emergent themes from the data. A team of two to three researchers will initially apply the codebook to two transcripts and review interrater reliability before moving forward to coding the remaining transcripts. The research team will meet regularly to discuss the coding process and ensure alignment and agreement. Data will be coded and analyzed to better understand the types and structures of incentives that motivate VHTs, the community’s relationship and engagement with VHTs, and community perceptions of VHT activities in advancing health, sanitation, and quality of care services. Data will be coded using QSR’s NVivo software [28].

### Data monitoring and management

Members of the study team will be responsible for reviewing incoming data. The study team does not anticipate any harm to be caused to study participants because of the intervention. Data from the VHT survey and VHT phone survey will be collected using ODK, a secure data collection platform. Any paper forms collected in the study will be kept in locked and secure storage cabinets, with only requisite personnel having access. Any data that has personally identifiable information will be kept on a secure server.

## Discussion

This study evaluates the impact of a public recognition intervention on VHT motivation, performance, and retention in Uganda. Given the limited financial support for the VHT program, the intervention was designed—through stakeholder engagement—with an emphasis on incentives that can be sustained beyond the context of this study and that are relatively inexpensive to administer. The intention of this study is not to suggest possible alternatives to financial compensation, but rather, additions to financial compensation that can be sustained locally.

As this study is being implemented during the ongoing COVID-19 pandemic, various COVID-19-related service disruptions may occur during the study. We expect that regular contact with the VHTs via phone calls will enable the research team to monitor these disruptions and account for them in the analyses and interpretation of the study results. The study predominantly relies upon VHT self-reporting data, which are subject to reporting and desirability bias; we plan on triangulating the data from multiple sources. There may be intervention spillover between the intervention and comparison parishes; however, VHTs in the comparison parishes will not be eligible for public recognition. This may lead to VHTs in the comparison parishes having lowered job satisfaction or motivation given that they are ineligible for the intervention. Lastly, incentives such as social recognition take time to register and require time to see corresponding improvements as a result of the incentives. Our study period is 12 months, which allows for four rounds of incentives to be delivered; this may be insufficient in capturing the longer-term effect of the intervention.

## Dissemination

Dissemination will include sharing findings through workshops, presentations, and peer-reviewed journal articles. We will work with District-level stakeholders and policymakers to disseminate and translate research findings to continue to inform the VHT policy in Uganda.

## Trial status

This is the final study protocol as of 17 November 2022. Study recruitment began in August 2021 and is expected to be completed as of March 2023.

## Supplementary Information

**Additional file 1.** SPIRIT checklist.

**Additional file 2.** VHT/ICCM Quarterly Summary.

**Additional file 3.** Consent forms.

## Data Availability

Data can be made available on request after the initial analysis is completed and disseminated.

## Abbreviations

CHW: Community health worker
DCE: Discrete choice experiment
DHO: District Health Officers
DiD: Difference-in-differences analysis
FGD: Focus group discussion
GEE: Generalized Estimating Equations
ITT: Intention-to-treat
ODK: Open Data Kit
PHC: Primary healthcare
RCT: Randomized controlled trial
VHT: Village health team
VHW: Village health worker
