# Supplementary material for: Impact of a recognition package as an incentive to strengthen the motivation, performance, and retention of village health teams in Uganda: a study protocol for a cluster randomized controlled trial
Source: Trials. 2023 Jun 23;24:428. doi: 10.1186/s13063-023-07426-6 (PMC10288687; doi:10.1186/s13063-023-07426-6)
Supplement: Supplementary file 2 — Additional file 2. VHT/ICCM Quarterly Summary. [file 13063_2023_7426_MOESM2_ESM.pdf]

MINISTRY OF HEALTH

(HMIS Form 097b)
VHT/ICCM Quarterly Village Report

Health Unit Name: .... Level: ....CODE..................

District/HSD Name:...

Financial Year: ....

(Print Version September 2019)

HMIS 097b: VHT/ICCM QUARTERLY VILLAGE REPORT

DESCRIPTION AND INSTRUCTIONS

Objective: To summarize information collected in the household summary by individual VHTs by Village

Timing: Quarterly

Copies: **Two Copies (Carbonized).** Original copy is submitted to Health Facility and a copy is retained at the Village with the VHT team leader.

Responsibilities: VHT Team Leader.

PROCEDURE:

1. The VHT/ICCM quarterly report form will have; the reporting months of the quarter and year, the name of the village, the Parish, Sub County, the Health Sub-District and the District. It has a part for the name and title of the person reporting and the one receiving the reports respectively.
2. It summaries the data variables from the Quarterly Household summary.
3. Just below the form, the VHTs compile the quarterly VHT/ICCM village report which also captures information on general observations like, disease outbreaks
4. The data is disaggregated under male and female and in some sections totals are captured.
5. It is very important that the VHT members summarize and submit their quarterly VHT/ICCM Summary, because of the following reasons:
    - Health workers at the health unit will know about births, deaths, illnesses, and other important health information about the village.
    - It enables the health unit plan for ways of improving health services in the village
    - It enables the health unit know what activities the VHT is has carried out

HMIS 097b: VHT/ICCM QUARTERLY SUMMARY Page 1

MINISTRY OF HEALTH

VHT/ICCM QUARTERLY REPORT

Reporting Period (Months): ……………………………………………………………………………

Village ……………………………………………………………………………

Parish ……………………………………………………………………………

Health Facility: ……………………………………………………………………………

Sub-County: ……………………………………………………………………………

Health-Sub-District: ……………………………………………………………………………

District: ……………………………………………………………………………

Name &Signature of VHT Village Team Leader: ……………………………………………

Name, Title & Signature of Person Receiving the Report: ……………………………………

Date Received: ……………………………………………………………………………

SUMMARY REPORT

VS 01 Number of Households……………………………………………………………

VS 02 Number of VHTs implementing ICCM……………………………………………

VS 03 Number of VHTs implementing Family Planning………………………………

VS 04 Number of VHTs attached to the facility that submitted a report (All programs)……………

VS 05 Number of VHTs expected to report……………………………………………

VS 06 Number of villages reporting ……………………………………………………

SN	PARAMETER		MALE	FEMALE	TOTAL
**SECTION A: VHT**					
**VH01**	Number of children less than 1 month				
**VH02**	Number of children died less than 1 month				
**VH03**	Number of children 1 to 11months				
**VH04**	Number of children died 1 to11months				
**VH05**	Number of children 1yr to 5yrs				
**VH06**	Number of children died 1yr to 5yrs				
**VH07**	Number of children under 5 years				
**VH08**	Number of children under 5yrs received vitamin A in last 6 months				
**VH09**	Number of children under 5yrs dewormed in the last 6 months				
**VH10**	Total Number of Children Under 5 yrs screened using MUAC				
**VH11**	Total Number of Children Under 5 yrs with;	Yellow MUAC			
		Red MUAC			
		Oedema			
**VH12**	Total number of children under 5 yrs with oedema, red or yellow MUAC referred to a health facility for care				
**VH13**	Total number of referred children under 5 yrs with oedema, red or yellow MUAC followed up and were linked to care				
**VH14**	Total Number of Pregnant/Lactating Women screened using MUAC				
**VH15**	Total Number of Pregnant/ Lactating Women with;	Yellow MUAC			
		Red MUAC			

Print Version September 2019

HMIS 097b: VHT/ICCM QUARTERLY SUMMARY Page 2

SN	PARAMETER	MALE	FEMALE	TOTAL
VH16	Total number of Pregnant/Lactating women with red or yellow MUAC referred to a health facility for care			
VH17	Total Number of referred Pregnant/Lactating Women with red or yellow MUAC followed up and were linked to care			
VH18	Number of children under 5yrs with Up to Date immunisation			
VH19	Number of persons with disability			
VH20	Number of HIV positive patients seen in the community			
VH21	Number of HIV/AIDS patients not on ART			
VH22	Number of TB cases identified in the community			
VH23	Number of TB cases that are not on TB Treatment in households			
VH24	Number of people using Family Planning services (information & methods)			
VH25	Number of deliveries at home			
VH26	Number of women who died during pregnancy			
VH27	Number of women who attended at least 4 ANC visits			
VH28	Number of women who attended at least 8 ANC visits			
VH29	Number of adolescents and young people (10-19 & 20-24 years)			
VH30	Number of adolescents and young people who received HPV vaccine			
VH31	Number of adolescents and young people who received TD vaccine			
VH32	Total Number of Household members using LLIN			
VH33	Number of households with latrines			
VH34	Number of households with improved latrines			
VH35	Number of households with hand washing facilities			
VH36	Number of households with safe drinking water			
VH37	Number of households that are open defecation free			
**SECTION B: ICCM**				
IC01	Total Number of sick Children 2 months – 5 years seen/attended to by the VHT			
IC02	Total Number of sick Children 2 months – 5 years with Diarrhea			
IC03	Number of Sick children 2 months - 5yrs confirmed Diarrhea cases that received ORS- Zinc co-pack			
IC04	Number of Children 2 months - 5yrs seen by VHT and treated within 24hrs for Diarrhoea			
IC05	Number of Children 2 months - 5yrs cases with fever seen by VHT_s			
IC06	Number of Children 2months - 5yrs cases with fever that received malaria RDT seen by VHT			
IC07	Number of Sick Children 2months-5yrs with confirmed Malaria by RDT			
IC08	Number of Sick children 2 months- 5yrs cases that received ACT			
IC09	Number of Sick children 2 months -5yrs with fever + danger signs seen in the community			
IC10	Number of Sick children 2 months - 5yrs with fever and danger signs treated with rectal artesunate			
IC11	Number of Sick children 2 months – 5 years seen by VHT and treated within 24hrs for fever			
IC12	Total Number of sick Children 2 months – 5 years with fast breathing / Pneumonia			
IC13	Number of Sick children 2 months - 5yrs confirmed pneumonia cases that received amoxicillin dispersible tablets			

HMIS 097b: VHT/ICCM QUARTERLY SUMMARY Page 3

SN	PARAMETER	MALE	FEMALE	TOTAL
**SECTION B: ICCM Ctd.....**				
**IC14**	Number of Sick children 2months - 5yrs seen by VHT and treated within 24hrs for pneumonia			
**IC15**	Number of Sick children who were managed by VHTs and recovered			
**IC16**	Number of children seen by VHTs that are exposed to HIV			
**IC17**	Number of children seen by VHTs with suspected TB			
**IC18**	Total Number of New Born babies visited twice in the first week of life by the VHT			
**IC19**	Number of sick children that were referred			
**Drug Availability and Stock Status**				
**SS01**	Total number of Villages with stock out of First line anti-Malarial (ACT)			
**SS02**	Total number of Villages with stock out of Amoxicillin dispersible			
**SS03**	Total number of Villages with stock out of ORS+ Zinc			
**SS04**	Total number of Villages with stock out of Misoprostol tablets			
**SS05**	Total number of Villages with stock out of Injectable DMPA			
**SS06**	Total number of Villages with stock out of Combined Oral Contraceptive			
**SS07**	Total number of Villages with stock out of Progestin only pills			
**SS08**	Total number of Villages with stock out of Condoms			
**SS09**	Total number of Villages with stock out of RDTs			
**SS10**	Total number of Villages with stock out of Emergency Contraceptives			
**SS11**	Total number of Villages with stock out of (specify)			

General Observations noted in the Village for example disease outbreaks
