# Supplementary material for: Impact of a recognition package as an incentive to strengthen the motivation, performance, and retention of village health teams in Uganda: a study protocol for a cluster randomized controlled trial
Source: Trials. 2023 Jun 23;24:428. doi: 10.1186/s13063-023-07426-6 (PMC10288687; doi:10.1186/s13063-023-07426-6)
Supplement: Supplementary file 3 — Additional file 3. Consent forms. [file 13063_2023_7426_MOESM3_ESM.docx]

**Appendix 3: Model Consent Forms**

**Appendix 3.1: VHT Focus Group Discussion Consent**

We are a study team from [Makerere University/Johns Hopkins University] and would like to ask you a few questions about VHTs, their role, and ways to support VHT retention. This conversation will be done in a group setting. This study will generate evidence to help the Ministry of Health, develop policies to help strengthen and support the VHT program. We ask you to join our research study because you are a practicing VHT in Masindi District. You do not have to participate in this, it is your choice.

**Why is this research being done?**

This focus group discussion is a part of a study looking at how to help strengthen and support the VHT program in Uganda.

**What will happen if you join this study?**

If you say yes, we will ask you questions about your job preferences to understand the types and structure of incentives that support and motivate your work as VHTs. The goal of the discussion is to identify the range of incentives such as training, recognition, identification, etc. that might be valued by VHTs. The group discussion will take between 1-1.5 hours, and will be audio-recorded. If, at any point, you have any questions and/or concerns during this consent process, please stop me to ask.

**Who can join this study?**

You have been invited to participate in this study because you are a practicing VHT in the randomized intervention parishes in Masindi district.

**What happens to data that are collected in the study?**

The data we collect from you will help advance science and public health. As a participant, you will not own your research data, and you will not benefit financially from any new product or idea that might arise from our work.

Sharing of research data is often done to increase what scientists can learn. The data you provide us might be shared:

- Directly with other researchers, funders, government agencies, and/or publishers of papers.
- Through government or other databases/repositories.

We will do our best to protect the data you provide. Sharing of data would only be done anonymously, and will not be linked to your name or address. If you are not comfortable with the use of your data in this and/or future research, you may not want to participate in this study.

**What are the risks or discomforts of the study?**

There are minimal risks from participating in this study. A potential risk may be a breach of confidentiality – or, your information being shared to people outside of the research team. However, we request that all participants keep this discussion private. The research team does not plan to name any individuals who participated in the study. The information you share during your participation will be kept confidentially as possible.

Additionally, some questions might be of a sensitive topic to you. Please remember that you do not have to answer any questions that make you uncomfortable. You may stop at any time during the group discussion without any penalties or loss of any potential benefits.

**What are the potential benefits to being in the study?**

As your parish is receiving the intervention, there will be some direct benefits through receiving the incentive package. The exact benefits will be determined after these discussions. Additionally, the potential benefits of sharing results from this study can help strengthen VHT programs in the country and improve the health of your communities.

**How will the confidentiality of your data be protected?**

The research team will make effort to protect the privacy of participants and maintain confidentiality of all information provided by them. We will ensure that all data kept on paper will be managed by a limited number of Makerere University researchers before being stored in a locked cabinet in a secure location. All digital data will be password protected on secured computers. Identifying information will not be used in reports or presentations.

Access to raw data will be restricted to the research team for analysis and synthesis for use.

**What are your options if you do not want to be in the study?**

Your participation in this study is not mandatory.

**What are the costs and compensations for being a part of the study?**

You will receive a token monetary payment for your time and participation in the study.

**Collection of audio/visual images**

The group discussions you are participating in will be audio-recorded. These audio-recordings will be kept confidential and stored in a safe and secure location. Photographs may also be taken but it will just be of the group discussions, and to document the processes that have taken place. No full-face, person-specific photographs will be taken.

If you are not comfortable with this, you might not want to participate in this study.

**Questions about the study**

Do you have any questions? If you have a concern about any aspect of the study, you should ask to speak to the researchers. They will do their best to answer your questions.

**What is the Institutional Review Board (IRB) and how does it protect you?**

This study has been reviewed by an Institutional Review Board (IRB), a group of people including scientists and community people, that reviews human research studies. The IRB can help you if you have questions about your rights as a research participant, or if you have other questions, concerns, or complaints about the research study.

Call or contact the <<**Makerere University** >> if you have questions about your rights as a study participant. Contact the IRB if you feel you have not been treated fairly or if you have other concerns. The IRB contact information is:

Address: Dr. Suzanne Kiwanuka, The Chair of the Higher Degrees, Research and Ethics Committee (HDREC), Makerere University School of Public Health, P.O. Box 7072 – Kampala, Uganda

Telephone: 0772886377 / +256-393-291-387 Toll Free:

E-mail: skiwanuka@musph.ac.ug

**Statement of voluntariness**

Your participation in this study is completely voluntary. If you decide not to participate, you will not lose any existing benefits to which you are entitled. If you agree to participate in this study, you may end your participation at any time without penalty or loss of existing benefits to which you are entitled. If you decide to take part, you are free to not answer any questions. You are free to withdraw at any time without affecting your relationship with your supervisors or employers.

**Dissemination of results**

Any new information that has relevance to you as a research participant will be made available to you through local channels. The results, which will be anonymized and combined, will be discussed by the research team, implementing partners, and policymakers. Results and datasets may be presented at conferences, published in scientific journals, and used by other researchers. Findings from the study will be disseminated at the community and national levels.

**Ethical approval**

This study has been reviewed by the Chair of the Higher Degrees, Research, and Ethics Committee (HDREC) at Makerere University School of Public Health and the Johns Hopkins Bloomberg School of Public Health Institutional Review Board.

**Consent**

Statement of consent after understanding the study and signature portion.

........................................................................... has described to me what is going to be done, the risks, the benefits involved and my rights regarding this study. I understand that my decision to participate in this study will not alter my usual medical care. In the use of this information, my identity will be concealed. I am aware that I may withdraw at any time. I understand that by signing this form, I do not waive any of my legal rights but merely indicate that I have been informed about the research study in which I am voluntarily agreeing to participate. A copy of this form will be provided to me.

___________________________ ______________________ ________

Participant name Participant signature/thumb print Date

___________________________ ______________________ ________

Interviewer name Interviewer signature Date

Appendix 3.2 Community Leaders Focus Group Discussion Consent Form

**Key Information about the Study:**

We are a study team from [Makerere University/Johns Hopkins University] and would like to ask you a few questions about VHTs, their role, and ways to support the VHT to improve their retention. This conversation will be done in a group setting. This study will generate evidence to help the Ministry of Health, with developing policies to help strengthen and support the VHT program. We ask you to join our research study because you are a community leader in Masindi District. You do not have to participate in this, it is your choice.

**Why is this research being done?**

This focus group discussion is a part of a study looking at how to help strengthen and support the VHT program in Uganda.

**What will happen if you join this study?**

If you say yes, we will ask you questions about your relationship and engagement with VHTs, and your perceptions on how VHTs are performing their responsibilities in advancing health, sanitation, and quality of care in the community. The goal of the discussion is to understand how we can support VHTs better. The group discussion will take between 1-1.5 hours, and will be audio-recorded. If, at any point, you have any questions and/or concerns during this consent process, please stop me to ask.

**Who can join this study?**

You have been invited to participate in this study because you are a community leader in Masindi district.

**What happens to data that are collected in the study?**

The data we collect from you will help advance science and public health. As a participant, you will not own your research data, and you will not benefit financially from any new product or idea that might arise from our work.

Sharing of research data is often done to increase what scientists can learn. The data you provide us might be shared:

- Directly with other researchers, funders, government agencies, and/or publishers of papers.
- Through government or other databases/repositories.

We will do our best to protect the data you provide. Sharing of data would only be done anonymously, and will not be linked to your name or address. If you are not comfortable with the use of your data in this and/or future research, you may not want to participate in this study.

**What are the risks or discomforts of the study?**

There are minimal risks from participating in this study. A potential risk may be a breach of confidentiality – or, your information being shared to people outside of the research team. However, we request that all participants keep this discussion private. The research team does not plan to name any individuals who participated in the study. The information you share during your participation will be kept confidentially as possible.

Additionally, some questions might be of a sensitive topic to you. Please remember that you do not have to answer any questions that make you uncomfortable. You may stop at any time during the group discussion without any penalties or loss of any potential benefits.

**What are the potential benefits to being in the study?**

There are no direct benefits from participating in this study. However, the potential benefits of sharing results from this study can help strengthen VHT programs in the country and improve the health of your communities.

**How will the confidentiality of your data be protected?**

The research team will make effort to protect the privacy of participants and maintain confidentiality of all information provided by them. We will ensure that all data kept on paper will be managed by a limited number of Makerere University researchers before being stored in a locked cabinet in a secure location. All digital data will be password protected on secured computers. Identifying information will not be used in reports or presentations.

Access to raw data will be restricted to the research team for analysis and synthesis for use.

**What are your options if you do not want to be in the study?**

Your participation in this study is not mandatory.

**What are the costs and compensations for being a part of the study?**

You will receive a token monetary payment for your time and participation in the study.

**Collection of audio/visual images**

The group discussions you are participating in will be audio-recorded. These audio-recordings will be kept confidential and stored in a safe and secure location. Photographs may also be taken but it will just be of the group discussions, and to document the processes that have taken place. No full-face, person-specific photographs will be taken.

If you are not comfortable with this, you might not want to participate in this study.

**Questions about the study**

Do you have any questions? If you have a concern about any aspect of the study, you should ask to speak to the researchers. They will do their best to answer your questions.

**What is the Institutional Review Board (IRB) and how does it protect you?**

This study has been reviewed by an Institutional Review Board (IRB), a group of people including scientists and community people, that reviews human research studies. The IRB can help you if you have questions about your rights as a research participant, or if you have other questions, concerns, or complaints about the research study.

Call or contact the <<**Makerere University** >> if you have questions about your rights as a study participant. Contact the IRB if you feel you have not been treated fairly or if you have other concerns. The IRB contact information is:

Address: Dr. Suzanne Kiwanuka, The Chair of the Higher Degrees, Research and Ethics Committee (HDREC), Makerere University School of Public Health, P.O. Box 7072 – Kampala, Uganda

Telephone: 0772886377 / +256-393-291-387 Toll Free:

E-mail: skiwanuka@musph.ac.ug

**Statement of voluntariness**

Your participation in this study is completely voluntary. If you decide not to participate, you will not lose any existing benefits to which you are entitled. If you agree to participate in this study, you may end your participation at any time without penalty or loss of existing benefits to which you are entitled. If you decide to take part, you are free to not answer any questions. You are free to withdraw at any time without affecting your relationship with your supervisors or employers.

**Dissemination of results**

Any new information that has relevance to you as a research participant will be made available to you through local channels. The results, which will be anonymized and combined, will be discussed by the research team, implementing partners, and policymakers. Results and datasets may be presented at conferences, published in scientific journals, and used by other researchers. Findings from the study will be disseminated at the community and national levels.

**Ethical approval**

This study has been reviewed by the Chair of the Higher Degrees, Research, and Ethics Committee (HDREC) at Makerere University School of Public Health and the Johns Hopkins Bloomberg School of Public Health Institutional Review Board.

**Consent**

Statement of consent after understanding the study and signature portion.

........................................................................... has described to me what is going to be done, the risks, the benefits involved and my rights regarding this study. I understand that my decision to participate in this study will not alter my usual medical care. In the use of this information, my identity will be concealed. I am aware that I may withdraw at any time. I understand that by signing this form, I do not waive any of my legal rights but merely indicate that I have been informed about the research study in which I am voluntarily agreeing to participate. A copy of this form will be provided to me.

___________________________ ______________________ ________

Participant name Participant signature/thumb print Date

___________________________ ______________________ ________

Interviewer name Interviewer signature Date

Appendix 3.3: VHT Survey and VHT Phone Survey Consent Form

**Key Information about the Study:**

We are a study team from [Makerere University/Johns Hopkins University] and would like to ask you a few questions about your work and role as a VHT. We will be asking some questions around your education and personal/family history. This conversation will be through a questionnaire provided twice, and a phone survey provided monthly, over the next 12 months.

**Why is this research being done?**

The questionnaire and phone survey are part of a study looking at incentive policies to help strengthen and support the VHT program in Uganda. We are testing the use of an incentive package at the parish-level in three counties within Masindi district. The phone survey is to better understand your work and responsibilities as a VHT over the past three months. The evidence generated by this study will help the Ministry of Health, international donors, and implementing partners to develop policies to support the VHT program.

**Who can join this study?**

We are asking you to join our research study because you are a practicing VHT working in Masindi district. You do not have to participate in this, as it is your choice.

**What will happen if you join this study?**

If you say yes, you are consenting to participate in the questionnaire and the phone survey. Your responses will be digitally recorded. For the questionnaire, we will ask you questions about your current work and responsibilities as a VHT, your background and training, your motivation, and your experience during COVID-19. The questionnaire occurs twice, at the beginning of the study and at twelve months and will take between 45-60 minutes; your answers will be recorded.

For the phone survey, we will ask you questions related to your work as a VHT over the past month. The survey will occur monthly OR twelve times over 12 months, and each survey will take between 10-15 minutes. Your answers will be digitally recorded. If, at any point, you have any questions and/or concerns during this consent process, please stop me to ask.

**What happens to data that are collected in the study?**

The data we collect from you will help advance science and public health. As a participant, you will not own your research data, and you will not benefit financially from any new product or idea that might arise from our work.

Sharing of research data is often done to increase what scientists can learn. The data you provide us might be shared:

- Directly with other researchers, funders, government agencies, and/or publishers of papers.
- Through government or other databases/repositories.

We will do our best to protect the data you provide. Sharing of data would only be done anonymously, and will not be linked to your name or address. If you are not comfortable with the use of your data in this and/or future research, you may not want to participate in this study.

**What are the risks or discomforts of the study?**

There are minimal risks from participating in this study. A potential risk may be a breach of confidentiality – or, your information being shared to people outside of the research team. The research team does not plan to name any individuals who participated in the study. The information you share during your participation will be kept confidentially as possible, and in a secure and locked location.

Additionally, some questions might be of a sensitive topic to you, so please remember that you do not have to answer any questions that make you uncomfortable. You may stop at any time during the survey without any penalties or loss of any potential benefits.

**What are the potential benefits to being in the study?**

If your parish is receiving the intervention, there will be some direct benefits. The exact benefits are to be determined, but can include the following: changes in workload hours, measures of identification (e.g., badges, branded items), financial renumeration, etc.

If your parish is not receiving the intervention, there are no direct benefits from participating in this study. However, the potential benefits of sharing results from this study can help strengthen VHT programs in the country, and improve the health of your communities.

**How will confidentiality of your data be protected?**

The research team will make effort to protect the privacy of participants and maintain confidentiality of all information provided by them. We will ensure that all data kept on paper will be managed by a limited number of Makerere University researchers before being stored in a locked cabinet in a secure location. All digital data will be password protected on secured computers. Identifying information will not be used in reports or presentations.

Access to raw data will be restricted to the research team for analysis and synthesis for use.

**What are your options if you do not want to be in this study?**

Your participation in this study is not mandatory.

**What are the costs and compensations for being part of the study?**

You will receive a token monetary payment for your time and participation in the study.

**Questions about the study**

Do you have any questions? If you have a concern about any aspect of the study, you should ask to speak to the researchers. They will do their best to answer your questions.

**What is the Institutional Review Board (IRB) and how does it protect you?**

This study has been reviewed by an Institutional Review Board (IRB), a group of people including scientists and community people, that reviews human research studies. The IRB can help you if you have questions about your rights as a research participant, or if you have other questions, concerns, or complaints about the research study.

Call or contact the <<**Makerere University** >> if you have questions about your rights as a study participant. Contact the IRB if you feel you have not been treated fairly or if you have other concerns. The IRB contact information is:

Address: Dr. Suzanne Kiwanuka, The Chair of the Higher Degrees, Research and Ethics Committee (HDREC), Makerere University School of Public Health, P.O. Box 7072 – Kampala, Uganda

Telephone: 0772886377 / +256-393-291-387 Toll Free:

E-mail: skiwanuka@musph.ac.ug

**Statement of voluntariness**

Your participation in this study is completely voluntary. If you decide not to participate, you will not lose any existing benefits to which you are entitled. If you agree to participate in this study, you may end your participation at any time without penalty or loss of existing benefits to which you are entitled. If you decide to take part, you are free to not answer any questions. You are free to withdraw at any time without affecting your relationship with your supervisors or employers.

**Dissemination of results**

Any new information that has relevance to you as a research participant will be made available to you through local channels. The anonymized, aggregated results of the study will be discussed by the research team, implementing partners, and policymakers. Results and datasets may be presented at conferences, published in scientific journals, and used by other researchers. Findings from the study will be disseminated at the community and national levels.

**Ethical approval**

This study has been reviewed by the Chair of the Higher Degrees, Research and Ethics Committee (HDREC) at Makerere University School of Public Health and the Johns Hopkins Bloomberg School of Public Health Institutional Review Board.

**Consent**

Statement of consent after understanding the study and signature portion.

........................................................................... has described to me what is going to be done, the risks, the benefits involved and my rights regarding this study. I understand that my decision to participate in this study will not alter my usual medical care. In the use of this information, my identity will be concealed. I am aware that I may withdraw at any time. I understand that by signing this form, I do not waive any of my legal rights but merely indicate that I have been informed about the research study in which I am voluntarily agreeing to participate. A copy of this form will be provided to me.

___________________________ ______________________ ________

Participant name Participant signature/thumb print Date

___________________________ ______________________ ________

Interviewer name Interviewer signature Date
